# Association of Modified Geriatric Nutrition Risk Index and Handgrip Strength With Survival in Cancer: A Multi-Centre Cohort Study

**DOI:** 10.3389/fnut.2022.850138

**Published:** 2022-04-01

**Authors:** Hailun Xie, Guotian Ruan, Heyang Zhang, Qi Zhang, Yizhong Ge, Mengmeng Song, Xi Zhang, Shiqi Lin, Xiaoyue Liu, Yuying Liu, Xiaowei Zhang, Xiangrui Li, Kangping Zhang, Ming Yang, Meng Tang, Zengning Li, Hanping Shi

**Affiliations:** ^1^Department of Gastrointestinal Surgery, Beijing Shijitan Hospital, Capital Medical University, Beijing, China; ^2^Department of Clinical Nutrition, Beijing Shijitan Hospital, Capital Medical University, Beijing, China; ^3^Beijing International Science and Technology Cooperation Base for Cancer Metabolism and Nutrition, Beijing, China; ^4^Key Laboratory of Cancer FSMP for State Market Regulation, Beijing, China; ^5^Department of Clinical Nutrition, The First Affiliated Hospital of Hebei Medical University, Shijiazhuang, China

**Keywords:** nutrition, inflammation, handgrip strength, cancer, prognostic, modified geriatric nutrition risk index

## Abstract

**Background:**

This study aimed to explore the value of combining the modified geriatric nutrition risk index (mGNRI) and handgrip strength (HGS) in the prognosis assessment of cancer.

**Methods:**

This multicenter, prospective cohort study, enrolled 5,607 cancer patients from 27 medical centers across 17 provinces in China between June 2012 and December 2019. The primary outcome was overall survival. Secondary outcomes included the Karnofsky Performance Scale (KPS) score, Patient-Generated Subjective Global Assessment (PG-SGA) score, cachexia, and admission 90-day outcome. A composite prognostic score (mGNRI-HGS score) was developed based on the mGNRI and HGS. The Kaplan–Meier method was used to draw the survival curve, and log-rank analysis was used to estimate the survival rate. The Cox proportional hazards model was used to investigate the associations of the mGNRI, HGS or mGNRI-HGS score with risk of mortality among the cancer patients, adjusted for potential confounders.

**Results:**

A low mGNRI (HR = 0.99, 95%CI = 0.98–0.99, *p* < 0.001) and low HGS (HR = 0.99, 95%CI = 0.98–0.99, *p* = 0.001) were associated with an increased risk of mortality. A severe mGNRI-HGS score was independently associated with reduced survival. Compared with patients with normal scores, the risk of mortality among the patients with moderate and severe mGNRI-HGS scores was 28.8 and 13.3% higher, respectively. Even within the same pathological stage, it presented significant gradient prognostic stratification. Additionally, a low mGNRI-HGS score was also independently associated with a higher risk of low KPS (*p* < 0.001), high PGSGA (*p* < 0.001), cachexia (*p* < 0.001), and adverse admission 90-day outcome (*p* < 0.001).

**Conclusions:**

The mGNRI and HGS may be useful predictors of long-term prognosis in cancer patients. The combination of the two methods provides effective prognostic stratification for cancer patients and could predict physical frailty, malnutrition, and cachexia.

## Introduction

Cancer is a heavy burden, with morbidity and mortality rapidly increasing worldwide. Currently, it is one of the leading global causes of death, with an estimated 19.3 million new cases and nearly 10 million deaths in 2020. Of these, China ranks first in cancer incidence, with about 4.57 million cases, and first in mortality, with approximately three million deaths (1). The incidence of cancer increases sharply with age. With China's population aging, the burden of cancer will increase correspondingly in the future (2, 3). Therefore, there is an urgent need to find effective, simple, and universal prognostic assessment tools for cancer to help formulate optimal treatment strategies.

Systemic inflammation caused by host-tumor interaction is closely related to the occurrence and development of cancer, and is considered the seventh marker of cancer (4, 5). Also closely related to the development and clinical outcome of the disease is nutritional status. Malnutrition can lead to disease progression and is a main reason for poor treatment effectiveness (6, 7). Recently, a C-reactive protein (CRP)-based modified geriatric nutrition risk index (mGNRI) was developed and proved to be an effective tool for predicting the clinical outcome of esophageal cancer (8). As a combined indicator of systemic inflammation and nutrition, the mGNRI has broad potential for assessing the prognosis of patients with cancer.

Hand grip strength (HGS) of the dominant hand is an economical and effective anthropometric measure of muscle function. Since 2018, the European Working Group on Sarcopenia in Older People (EWGSOP) has recommended HGS as an important indicator for defining sarcopenia in clinical practice (9). In addition, low HGS is recommended as the standard for the definition of cancer cachexia (10). Assessment of HGS provides significant additional prognostic information for patients with cancer, and reduced HGS is considered to be related to deterioration in patient survival (11, 12).

The prognostic value of a single indicator for patients with cancer is still limited, and the combination of multiple indicators may be a good direction for development. The mGNRI represents the inflammatory and nutritional status of patients, and HGS reflects their physical status. Whether the combination of the two can provide further prognostic and therapeutic guidance for cancer patients is unclear. Therefore, this study aimed to explore the value of combining the mGNRI and HGS as a prognostic tool for cancer patients and to provide reference values to optimize prognosis assessment and treatment strategies.

## Materials and Methods

### Study Design and Population

This was a multicenter, prospective cohort study. The patients were part of the Investigation on Nutrition Status and its Clinical Outcome of Common Cancers (INSCOC) project, which included patients with cancer from 27 clinical medical centers across 17 provinces in China, from June 2012 to December 2019. In this study, eligible patients were 18 years of age and older with a histopathological or cytological diagnosis of cancer. We excluded patients who were admitted for <24 h, were younger than 18 years old, were unwilling or unable to participate because of cognitive impairment, or who did not have complete data available on CRP, albumin, height, weight, and HGS. The patients were prospectively followed up by professionals until the last follow-up date (30/10/2020) or the date of death for any reason, and the follow-up outcome was recorded in detail. Follow-up was performed through face-to-face inquiries or telephone interviews. All patient data were analyzed anonymously. All patients provided written consent. This study was approved by the ethics committees of all participating institutions.

### Data Acquisition and Definitions

Baseline sociodemographic information was obtained by well-trained professionals when the patients were admitted to the hospital, including age, sex, smoking history, alcohol history, family history of cancer, comorbidities (hypertension and diabetes), and anthropometric measurements [height, weight, body mass index (BMI)]. Blood serological parameters collected at baseline included white blood cell (WBC), neutrophil, lymphocyte, platelet, and red blood cell (RBC) counts, hemoglobin (Hb), CRP, and serum albumin. All serological tests were performed within a week of admission. Tumor information included the tumor site and tumor-node-metastasis (TNM) stage (American Joint Committee on Cancer staging System, 8th Edition). Treatments included surgery, radiotherapy, and chemotherapy.

According to previous measurement methods (13). the electronic Hand Grip Dynamometer (CAMRY, Model EH101, Guagndong, China) was used to measure the HGS of dominant hands. The patients held the dynamometer with maximum strength with the dominant hand, the test was repeated three times, and the maximum HGS was recorded. The HGS of patients was measured before antitumor therapy. The GNRI was calculated using the following formula: 1.489 × albumin (g/dL) + 41.7 × present body weight (kg)/ideal body weight (kg). mGNRI was calculated as: (1.489 / CRP in mg/dL) + [41.7 × present body weight (kg)/ideal body weight (kg)]. Based on previous research, (14) the Lorentz formula was used to calculate the ideal body weight, as follows: height (cm)-100—{[height (cm)−150]/4} for men and height (cm)-100—{[height (cm)−150]/2.5} for women. The current body weight/ideal body weight was considered to be 1 when the current weight exceeded the ideal weight (15).

The primary outcome was overall survival (OS), defined as the period from the date of pathological diagnosis of cancer to the date of death or the last follow-up. Secondary outcomes included the Karnofsky Performance Scale (KPS) score ( ≤ 70 indicating risk), the Patient-Generated Subjective Global Assessment (PG-SGA) score (≥4 indicating risk), cachexia, and admission 90-day outcome. The KPS and PG-SGA were assessed and recorded by trained staff at baseline. The diagnosis of cachexia was based on the internationally recognized definition and diagnostic criteria for cancer cachexia presented by Fearon et al. in the 2011 International Consensus on Cachexia (16), as follows: (1) weight loss >5% of starting body weight in the past 6 months without dieting; (2) BMI <20 kg/m^2^ and any degree of weight loss >2%; or (3) Skeletal muscle depletion was evident, as estimated by the mid upper-arm muscle area (men: <32 cm^2^, women: <18 cm^2^). Patients meeting one or more of the above criteria were diagnosed with cancer cachexia. The admission 90-day outcome was defined as survival outcome within 90 days of hospitalization for anticancer therapy.

### Statistical Analysis

Optimal stratification was used to determine the threshold of continuous mGNRI and GNRI using log-rank statistics. Given the significant difference in HGS between men and women, we used sex-specific optimal stratification to determine the optimal threshold of continuous HGS in men and women, respectively. The optimum thresholds for GNRI and mGNRI are 93 and 43, respectively (Supplementary Figure S2, GNRI, mGNRI). Low GNRI is defined as <93, while above 93 is considered a high GNRI. Low mGNRI is defined as <43, and an mGNRI above 43 is considered a high mGNRI. The sex-specific optimum thresholds for HGS are 16.1 kg for women and 22.0 kg for men (Supplementary Figure S2, HGS). Subsequently, low HGS was defined as HGS for males <22.0 kg, HGS for females <16.1 kg, and otherwise, it was considered high. A composite prognostic score was developed using mGNRI and HGS: mGNRI and HGS were assigned, low mGNRI and low HGS were scored as 1, and high mGNRI and high HGS were scored as 0. The two scores were then summed to construct the mGNRI-HGS score. We classified the mGNRI-HGS score into three groups, namely normal (score of 0), moderate (score of 1), and severe (score of 2). mGNRI-HGS score, mGNRI, and HGS were the exposures for the present analysis.

Baseline characteristics of the study population were presented as mean (standard deviation) or median (interquartile range) for continuous variables, and as number (percentage) for categorical variables. Differences between groups were analyzed using the Chi-square test, *t*-test, or Kruskal–Wallis test, as appropriate. We fitted three statistical models, which were adjusted for potential confounding factors such as sociodemographic, clinical, and pathological features: model (a) did not adjust for any confounding factors; model (b) adjusted for age, sex, BMI, and TNM stage; model (c) controlled for the same factors as model b, plus tumor type, surgery, radiotherapy, chemotherapy, hypertension, diabetes, smoking, drinking, and family history. Similar to previous studies (17), we constructed a restricted cubic spline to evaluate the relationship between continuous covariates and mortality in cancer patients in the different models. The time-dependent area under the receiver operating characteristic curve (AUC) was used to compare the predictive capacity of GNRI and mGNRI.

The Cox proportional hazards model was used to estimate the hazard ratios (HRs) and 95% confidence intervals (CIs) of cancer mortality per standard deviation (SD) change or quartile 2, 3, 4 (compared with quartile (1) in mGNRI and HGS, and was adjusted for potential confounders including age, sex, BMI, TNM stage, tumor type, surgery, radiotherapy, chemotherapy, hypertension, diabetes, smoking, drinking, and family history. Meanwhile, we did subgroup analysis by age, sex, BMI, etc., and tested the interaction of the exposure with these factors and their effects on the outcome.

We then used the Cox proportional hazards model to examine the association of per 1-unit change (1 vs. 0, 2 vs. 0) of the mGNRI-HGS score with cancer mortality, and then adjusted for the same covariates. In addition, we did subgroup analysis by tumor types and pathological stages to test the dose response relationship of the exposure on the outcome.

The Kaplan-Meier method and log-rank analysis were used to estimate the differences in outcomes of various mGNRI-HGS score. We conducted subgroup survival analysis based on different pathological stages and tumor types to test the universality of the model. Univariate and multifactor logistic regression models were used to assess the association of mGNRI, HGS, and mGNRI-HGS score with low KPS, high PGSGA, cachexia, and admission 90-day outcome, adjusted for different models. The discriminant index, including C-statistic, continuous net reclassification improvement (cNRI), and integrated discrimination improvement (IDI) were used to compare the prognostic prediction ability of prediction covariates and prognostic gain of the combined pathological stages. Finally, we randomly assigned the total population to “validation a” (3,927 cases) and “validation b” (1,680 cases) in a ratio of 7:3 based on computer-generated random numbers, in order to perform randomized internal validation of the constructed combination score. A two-sided *p*-value of <0.05 was considered statistically significant for all analyses. R software, version 4.0.5 was used for statistical analyses.

## Results

### Baseline Characteristics

This study included 5,607 cancer patients with complete data from multiple centers (Supplementary Figure S1), including 3,378 males and 2,229 females, with a mean age of 59.4 (11.3) years. Based on the established thresholds, there were 70.29% patients with high GNRI, 29.71% with low GNRI, 54.25% with high mGNRI, and 45.75% with low mGNRI. There were 55.68% and 44.32% patients with high and low HGS, respectively. Detailed information on baseline characteristics is presented in Supplementary Table S1. Low mGNRI and low HGS were statistically associated with poor physical condition (high age, low BMI), poor nutritional status (low albumin, low RBC count, and low Hb), high inflammatory status (high WBC, neutrophil, and platelet counts and low lymphocyte count), and advanced pathological stage. In addition, both low mGNRI and low HGS were associated with adverse outcomes, including prolonged hospital stay, high KPS, high PG-SGA, cachexia, and low survival rates.

### Comparison of Survival Curves for mGNRI and HGS

**Figure 1:**
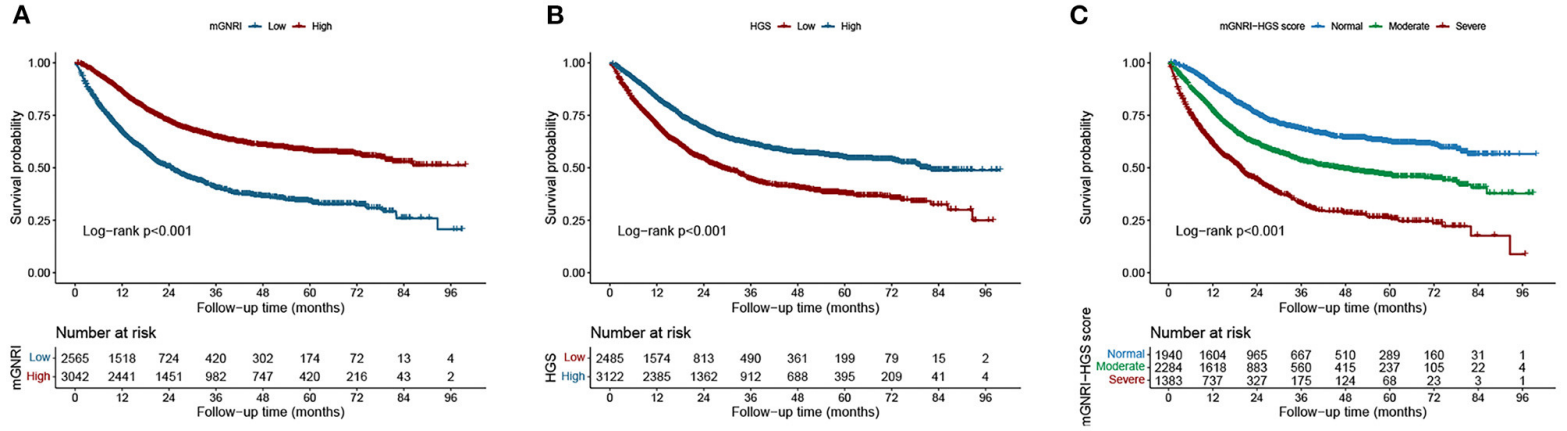
Kaplan-Meier curve of mGNRI, HGS, and mGNRI-HGS score in patients with cancer. **(A)**, mGNRI; **(B)**, HGS; **(C)**, mGNRI-HGS score.

We compared the effectiveness of mGNRI and GNRI in assessing the prognosis of cancer patients through AUC analysis (Supplementary Figure S3A), and the results showed that mGNRI was more effective than GNRI in predicting the prognosis of cancer patients in the total population and at various stages. In addition, compared to the GNRI, mGNRI performed better in stratifying the prognosis of cancer patients (Supplementary Figure S3B). The Kaplan–Meier survival curves revealed that a low mGNRI was associated with an increased risk of mortality in cancer patients. Compared with patients with a high mGNRI, patients with a low mGNRI had an approximately 19.72% (48.23 vs. 67.95%, log-rank *p* < 0.001) increased risk of death (Figure 1A). Patients with low HGS had an approximately 14.18% higher risk of death than those with high HGS (65.21 vs. 51.03%, log-rank *p* < 0.001) (Figure 1B). We further found that mGNRI and HGS can effectively stratify the prognosis of both male and female patients (Supplementary Figures S6A,B). It is worth noting that these differences were significant in different tumor types (lung cancer, gastrointestinal cancer, and non-gastrointestinal cancers) (Supplementary Figures S7A,B). In addition, mGNRI and HGS were also effective prognostic predictors in patients in various pathological stages (Supplementary Figures S8A,B).

### Relationship Between mGNRI and HGS and Survival of Patients

Restricted cubic spline plots suggested that mGNRI (Supplementary Figure S4A) and HGS (Supplementary Figure S4B) were significantly positively associated with patient prognosis. With decrease in the mGNRI and HGS, the prognosis of patients gradually worsened, and the trend was not affected by confounding factors. Both univariate and multivariable Cox proportional hazards models suggested that low mGNRI and low HGS were independent risk factors for prognosis (Supplementary Table S2). After adjusting for confounders, for every SD increase in the mGNRI and HGS, the risk of poor prognosis for cancer patients was reduced by 20% (HR = 0.80, 95% CI = 0.75–0.84, *p* < 0.001) and 16% (HR = 0.84, 95% CI = 0.80–0.88, *p* < 0.001), respectively (Supplementary Table S3).

In multivariate subgroup analysis, both the mGNRI and HGS were independent prognostic factors for the 32 patient subgroups (Supplementary Figures S5A,B). We found that low mGNRI was independently associated with low KPS (OR = 0.97, 95% CI = 0.96–0.98, *p* < 0.001), high PG-SGA (OR = 0.98, 95% CI = 0.97–0.98, *p* < 0.001), cachexia (OR = 0.99, 95% CI = 0.98–0.99, *p* < 0.001), and adverse admission 90-day outcomes (OR = 0.95, 95% CI = 0.92–0.97, *p* < 0.001), as was the case with low HGS (low KPS, OR = 0.93, 95% CI = 0.92–0.94, *p* < 0.001; high PG-SGA, OR = 0.97, 95% CI = 0.96–0.97, *p* < 0.001; Cachexia, OR = 0.98, 95% CI = 0.97–0.99, *p* < 0.001; adverse admission 90-day outcomes, OR = 0.95, 95% CI = 0.93–0.96, *p* < 0.001) (Supplementary Table S4).

### Construction of a Novel Score Based on mGNRI and HGS

Our results showed that mGNRI and HGS have marked value and relatively consistent weight in evaluating the adverse prognosis of cancer patients. Therefore, we developed a combination score using the mGNRI and HGS indexes. In the analysis of differences between groups, high mGNRI-HGS scores were closely associated with poor physical condition, poor nutritional status, high inflammatory status, and progressive pathological stage (Supplementary Table S5). Compared with patients with normal scores, the mortality risk of patients with moderate and severe scores was 28.8 and 13.3% higher, respectively (Figure 1C). In subgroup analysis by sex, higher mGNRI-HGS scores were still associated with reduced survival (Supplementary Figure S6). Different types of tumors failed to change the correlation between the mGNRI-HGS score and the prognosis of cancer patients (Supplementary Figure S7C). Notably, even in the same pathological stage, the mGNRI-HGS score presented significant gradient prognostic stratification (Supplementary Figure S8C), indicating that the score can further predict prognosis and stratify risk in patients at the same pathological stage.

**Figure 2:**
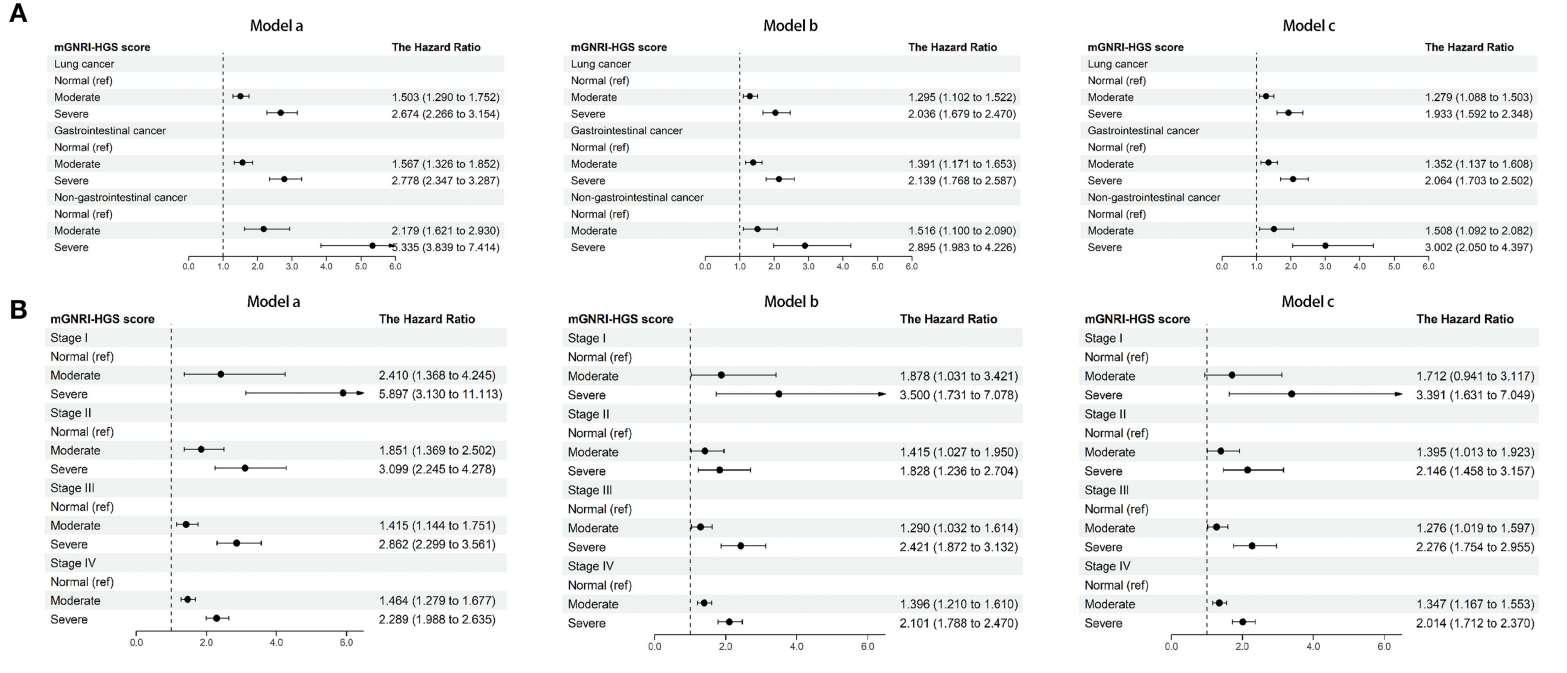
Dose-response effects of mGNRI-HGS score based on subgroup. **(A)**, tumor types; **(B)**, pathological stages. Model a, No adjusted. Model b, Adjusted for age, sex, BMI, TNM stage. Model c, Adjusted for age, sex, BMI, TNM stage, tumor type, surgery, radiotherapy, chemotherapy, hypertension, diabetes, smoking, drinking, family history.

**Figure 3:**
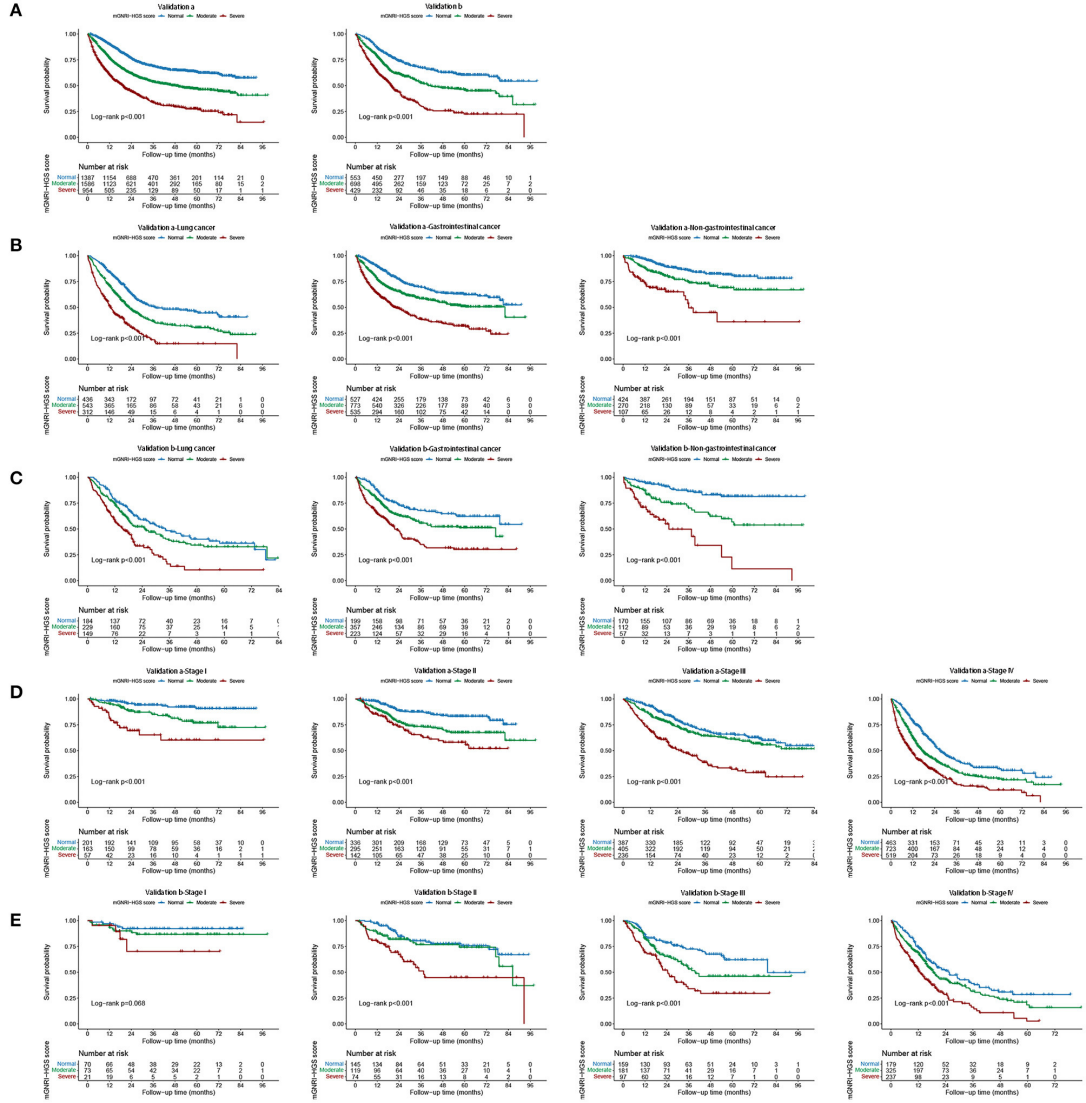
Internal validation of mGNRI-HGS score based different tumor types and pathological stages. **(A)** Internal validation of mGNRI-HGS score; **(B)** Internal validation a of mGNRI-HGS score based on tumor types; **(C)** Internal validation b of mGNRI-HGS score based on tumor types; **(D)** Internal validation a of mGNRI-HGS score based on pathological stages; **(E)** Internal validation b of mGNRI-HGS score based on pathological stages.

In multivariable Cox regression analysis, the mGNRI-HGS score remained independently associated with reduced survival (Table 1). For each change per 1-unit, the corresponding risk of adverse outcome increased by more than 37%. Compared with the normal group, the risk of adverse outcome in the severe group was more than doubled. In subgroup analysis, we found that the mGNRI-HGS score showed significant dose-response effects (Figures 2A,B). With increasing mGNRI-HGS scores, the risk of poor prognosis in the normal, moderate, and severe groups gradually increased. On multivariable logistic regression analysis, the severe mGNRI-HGS score was independently associated with an increased risk of low KPS, high PG-SGA, cachexia, and adverse admission 90-day outcome (Table 2). In comparative analysis with sub-components, the mGNRI-HGS score showed a great advantage in both prediction accuracy and gain for pathological stage (Supplementary Table S6). The randomized internal validation showed that the mGNRI-HGS score could still effectively stratify the prognosis of patients in the total population (Figure 3A), different tumor types (Figures 3B,C), and different pathological stages (Figures 3D,E).

**Table 1:** Trend test of the relationship between mGNRI-HGS score and survival.

**mGNRI-HGS score**	**Model a**	***p-*value**	**Model b**	***p-*value**	**Model c**	***p-*value**
Normal	Ref		Ref		Ref	
Moderate	1.744 (1.570, 1.937)	<0.001	1.413 (1.265, 1.578)	<0.001	1.368 (1.224, 1.527)	<0.001
Severe	3.171 (2.842, 3.537)	<0.001	2.203 (1.94, 2.502)	<0.001	2.153 (1.895, 2.447)	<0.001
*p* for trend		<0.001		<0.001		<0.001

*Model a, No adjusted; Model b, Adjusted for age, sex, BMI, TNM stage; Model c, Adjusted for age, sex, BMI, TNM stage, tumor type, surgery, radiotherapy, chemotherapy, hypertension, diabetes, smoking, drinking, family history*.

**Table 2:** Logistic regression analysis of mGNRI-HGS score associated with secondary outcome.

**KPS**						
**mGNRI-HGS score**	**Model a**	***p-*** **value**	**Model b**	***p-*** **value**	**Model c**	***p-*** **value**
Normal	Ref		Ref			
Moderate	3.738 (2.860, 4.885)	<0.001	3.271 (2.476, 4.322)	<0.001	3.275 (2.470, 4.343)	<0.001
Severe	8.581 (6.572, 11.204)	<0.001	6.499 (4.803, 8.793)	<0.001	6.326 (4.651, 8.603)	<0.001
*p* for trend		<0.001		<0.001		<0.001
**PGSGA**						
Normal	Ref		Ref		Ref	
Moderate	2.200 (1.945, 2.489)	<0.001	1.797 (1.573, 2.053)	<0.001	1.793 (1.568, 2.051)	<0.001
Severe	5.723 (4.880, 6.713)	<0.001	3.703 (3.088, 4.441)	<0.001	3.702 (3.082, 4.446)	<0.001
*p* for trend		<0.001		<0.001		<0.001
**Cachexia**						
Normal	Ref		Ref		Ref	
Moderate	2.252 (1.948, 2.603)	<0.001	1.700 (1.453, 1.988)	<0.001	1.697 (1.450, 1.987)	<0.001
Severe	4.356 (3.725, 5.094)	<0.001	2.448 (2.030, 2.953)	<0.001	2.434 (2.016, 2.939)	<0.001
*p* for trend		<0.001		<0.001		<0.001
**Admission 90 days outcome**						
Normal	Ref		Ref			
Moderate	4.381 (2.883, 6.656)	<0.001	3.099 (2.013, 4.770)	<0.001	2.942 (1.907, 4.537)	<0.001
Severe	11.422 (7.586, 17.199)	<0.001	6.301 (4.011, 9.898)	<0.001	5.803 (3.684, 9.140)	<0.001
*p* for trend		<0.001		<0.001		<0.001

*Model a, No adjusted; Model b, Adjusted for age, sex, BMI, TNM stage; Model c, Adjusted for age, sex, BMI, TNM stage, tumor type, surgery, radiotherapy, chemotherapy, hypertension, diabetes, smoking, drinking, family history*.

## Discussion

In the present study, we found that the mGNRI-HGS score could comprehensively reflect the physical condition, inflammatory state, and pathological characteristics of patients in this cohort. The mGNRI-HGS score proved to be an independent prognostic factor for cancer patients; as the mGNRI-HGS score increased, patient survival showed a step-like decline. The mGNRI-HGS score effectively differentiated outcomes in patients with the same pathological stage and presented a significant dose-response relationship, indicating that the score can be a useful prognostic index for tumor-related factors, independent of pathological stage. In addition, we found that the mGNRI-HGS score was an effective prognostic tool for different tumor types, suggesting that this comprehensive score can be used for prognosis assessment of different cancer populations. To further validate the effectiveness of the score, we conducted a randomized internal validation. The results showed that the mGNRI-HGS score was still an independent prognostic predictor for cancer and could effectively stratify the prognosis of cancer patients.

The mGNRI combines serological and anthropometric indicators to comprehensively reflect the patient's inflammatory and nutritional status. It was developed from the GNRI and emphasizes the role of CRP as a indicator of systemic inflammation (8). Systemic inflammation plays a key role in the development and progression of cancer. It stimulates angiogenesis and cell proliferation through induction of reactive oxygen and nitrogen species (18, 19). Serum CRP is the most representative marker of systemic inflammation in clinical practice. With inflammation, the liver inhibits the synthesis of albumin and promotes the synthesis of acute-phase proteins. However, albumin is easily affected by the fluid balance in the body, leading to instability, (20, 21) while CRP is widely regarded as an effective indicator of systemic inflammation given its stability (22). In this study, we found that compared with the GNRI, the mGNRI had a better predictive ability for the prognosis of cancer patients and performed better in stratifying the adverse risks of patients, which may be because of the ability of CRP in reflecting systemic inflammation. Since albumin instability may reduce its prognostic predictive ability in cancer patients, we chose CRP-based mGNRI to construct the prognostic score in this study.

HGS is a simple and effective method to assess the physical status of cancer patients. Low HGS has been shown to reflect poor prognosis in cancer patients (11, 23). Some studies have suggested that decreased muscle function in cancer patients is the result of local muscle inflammation, and that increased inflammatory cytokines can also lead to insulin resistance and muscle depletion by activating the ubiquitin-proteasome proteolytic pathway (24, 25). A decrease in muscle mass and strength can lead to changes in functional status, leading to limitations in daily activities. Low HGS is considered an external sign of decreased muscle function, and a low mGNRI reflects a high level of cancer-related inflammation (8, 26). In this study, we found that patients with both low mGNRI and low HGS had a more than 5-fold higher risk of functional decline compared to patients with normal results. The strong combination of the two may provide a reference for prognostic stratification of cancer and the choice of therapeutic strategies. As mGRNI and HGS have the advantages of simple operation and low price, the mGNRI-HGS score can be routinely measured in clinical practice for prognostic assessment of cancer patients, which has broad clinical application prospects.

The interaction between the tumor and the patient's local response has a profound impact on the patient's general condition, including on daily activities and nutritional status. Nutritional disorders caused by cancer also affect the outcome of cancer treatment, increasing the risk of infection and complications and reducing the efficacy and continuity of chemotherapy and radiotherapy (6, 27). We further found that the mGNRI and HGS were useful indicators of malnutrition, cachexia, and short-term outcomes in cancer patients, and that the combination of the two could significantly enhance prediction of the risk of adverse outcomes.

The purpose of this study was to provide routes for early detection of the adverse state of cancer patients, evidence on tools for assessing the prognosis of cancer patients, and references for formulating treatment strategies for cancer patients through the comprehensive evaluation of anthropometric measurements and serum biological indicators. However, we note a few limitations that should be considered. First, although internal validation was conducted and good consistency was achieved, it is still necessary to validate our results with a larger sample and multi-center external cohort in the future. Second, the data on inflammatory nutritional indicators and body measurements were only evaluated at a single time point, failing to reflect the impact of their trajectory changes on prognosis, which needs to be further explored in the future. Finally, this study only included Chinese patients, and its extension to populations in other countries remains to be explored.

## Conclusion

This study demonstrated that the mGNRI is a useful indicator of long-term prognosis in cancer patients. The combination of mGNRI and HGS could provide effective prognostic stratification for cancer patients and predict physical frailty, malnutrition, and cachexia.

## Data Availability Statement

The original contributions presented in the study are included in the article/Supplementary Materials, further inquiries can be directed to the corresponding author.

## Ethics Statement

The studies involving human participants were reviewed and approved by the Institutional Review Board of each hospital (Registration Number: ChiCTR1800020329). This study followed the Helsinki declaration. All participants signed an informed consent form to participate in this study.

## Author Contributions

HS, HX, GR, and HZ conceived and designed the study. HX, QZ, YG, MS, XiZ, XLiu, and SL assisted with the development of the methods. HS, HX, GR, QZ, XiaZ, XLi, and KZ did the data analysis. MY, MT, ZL, and HX drafted the initial manuscript. HS is the guarantor and attests that all listed authors meet authorship criteria and that no others meeting the criteria have been omitted. All authors assisted with the interpretation of the findings, commented on drafts of the manuscript, and approved the final version.

## Funding

This study was supported by the National Key Research and Development Program to HS (No. 2017YFC1309200) and the Beijing Municipal Science and Technology Commission (SCW2018-06).

## Conflict of Interest

The authors declare that the research was conducted in the absence of any commercial or financial relationships that could be construed as a potential conflict of interest.

## Publisher's Note

All claims expressed in this article are solely those of the authors and do not necessarily represent those of their affiliated organizations, or those of the publisher, the editors and the reviewers. Any product that may be evaluated in this article, or claim that may be made by its manufacturer, is not guaranteed or endorsed by the publisher.

## Supplementary Material

Supplementary Figure S1 Study design.

Supplementary Figure S2 The optimum thresholds of mGNRI and HGS.

Supplementary Figure S3 Comparison the effectiveness of mGNRI and GNRI in predicting the prognosis of cancer patients. **(A)** The AUC of mGNRI and GNRI. **(B)** The survival curve of mGNRI and GNRI.

Supplementary Figure S4 The association between mGNRI and HGS and all-cause mortality in patients with cancer. **(A)**, Mgnri; **(B)**, HGS. Model a: No adjusted. Model b: Adjusted for age, sex, BMI, TNM stage. Model c: Adjusted for age, sex, BMI, TNM stage, tumor type, surgery, radiotherapy, chemotherapy, hypertension, diabetes, smoking, drinking, family history.

Supplementary Figure S5 The association between mGNRI and HGS and hazard risk of overall survival in various subgroups. (The model adjusted for age, sex, BMI, TNM stage, tumor type, surgery, radiotherapy, chemotherapy, hypertension, diabetes, smoking, drinking, family history). **(A)**, mGNRI; **(B)**, HGS.

Supplementary Figure S6 Stratified survival analysis of mGNRI, HGS, and mGNRI-HGS score based on sex. **(A)**, male; **(B)**, female.

Supplementary Figure S7 Stratified survival analysis of mGNRI, HGS, and mGNRI-HGS score based on tumor types. **(A)**, mGNRI; **(B)**, HGS; **(C)**, mGNRI-HGS score.

Supplementary Figure S8 Stratified survival analysis of mGNRI, HGS, and mGNRI-HGS score based on pathological stages. **(A)**, mGNRI; **(B)**, HGS; **(C)**, mGNRI-HGS score.

Supplementary Table S1 Association between the mGNRI, HGS and clinical characteristics.

Supplementary Table S2 Cox regression analysis of characteristics associated with overall survival.

Supplementary Table S3 Trend test of the relationship between mGNRI and HGS and survival.

Supplementary Table S4 Logistic regression analysis of mGNRI and HGS associated with secondary outcome.

Supplementary Table S5 Association between the mGNRI-HGS score and clinical characteristics.

Supplementary Table S6 Comparative analysis of the discrimination of mGNRI, HGS, mGNRI-HGS score for all-cause mortality in patients with cancer.
