# Supplementary figures and images for: Association of Modified Geriatric Nutrition Risk Index and Handgrip Strength With Survival in Cancer: A Multi-Centre Cohort Study
Source: Front Nutr. 2022 Apr 1;9:850138. doi: 10.3389/fnut.2022.850138 (PMC9012584; doi:10.3389/fnut.2022.850138)

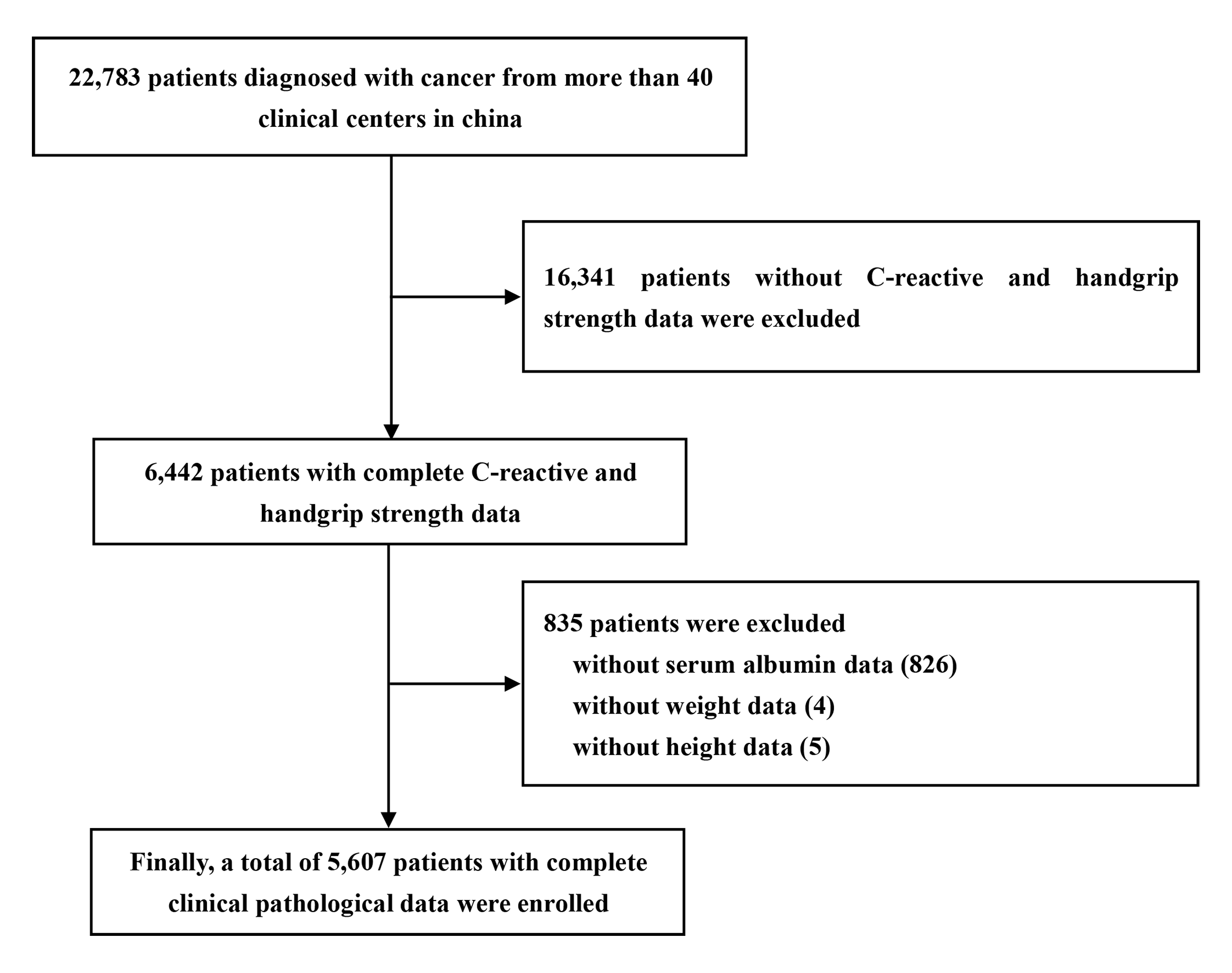

Supplement: Supplementary Figure S1 — Study design. [file Image_1.TIF]

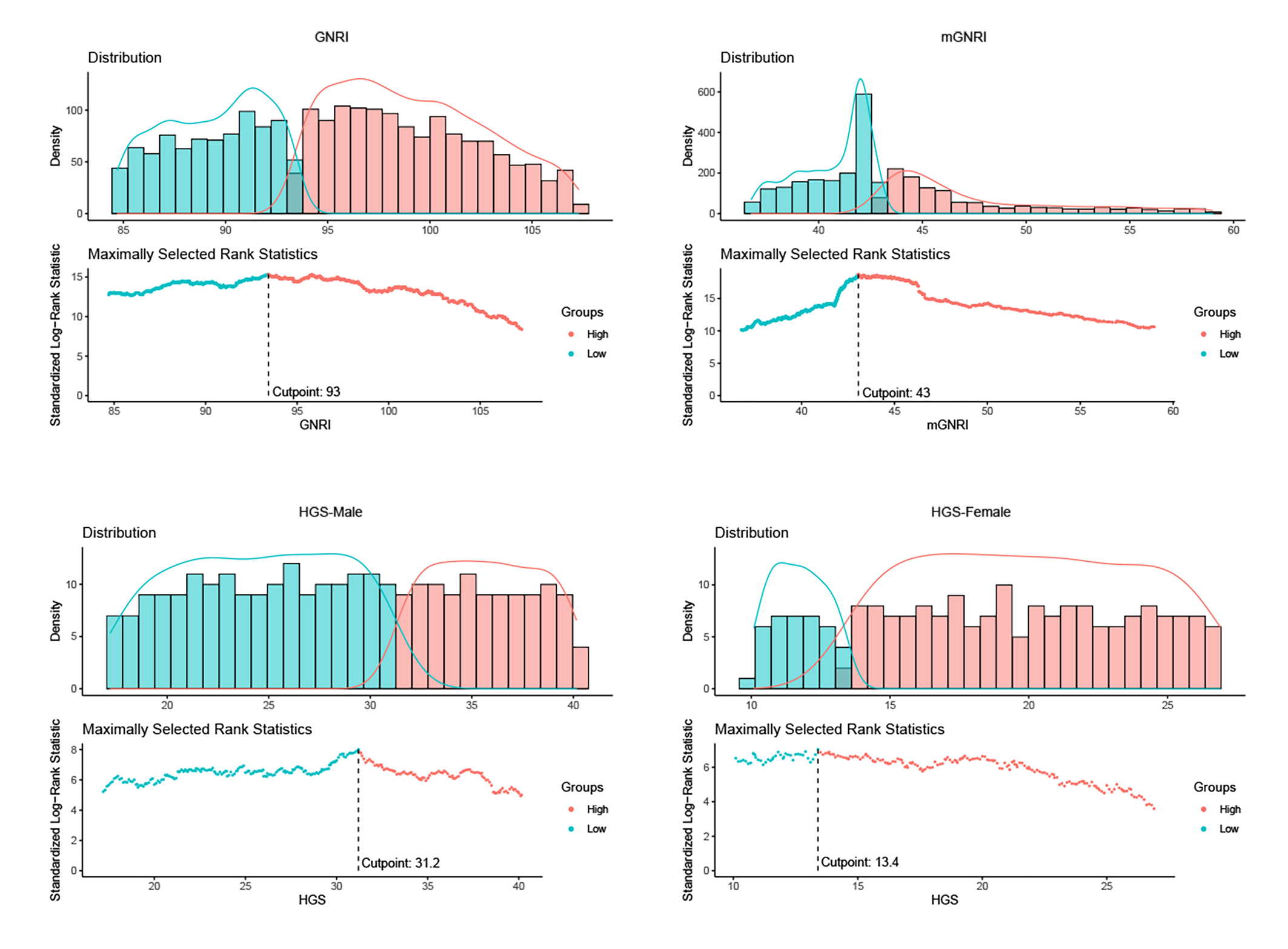

Supplement: Supplementary Figure S2 — The optimum thresholds of mGNRI and HGS. [file Image_2.TIF]

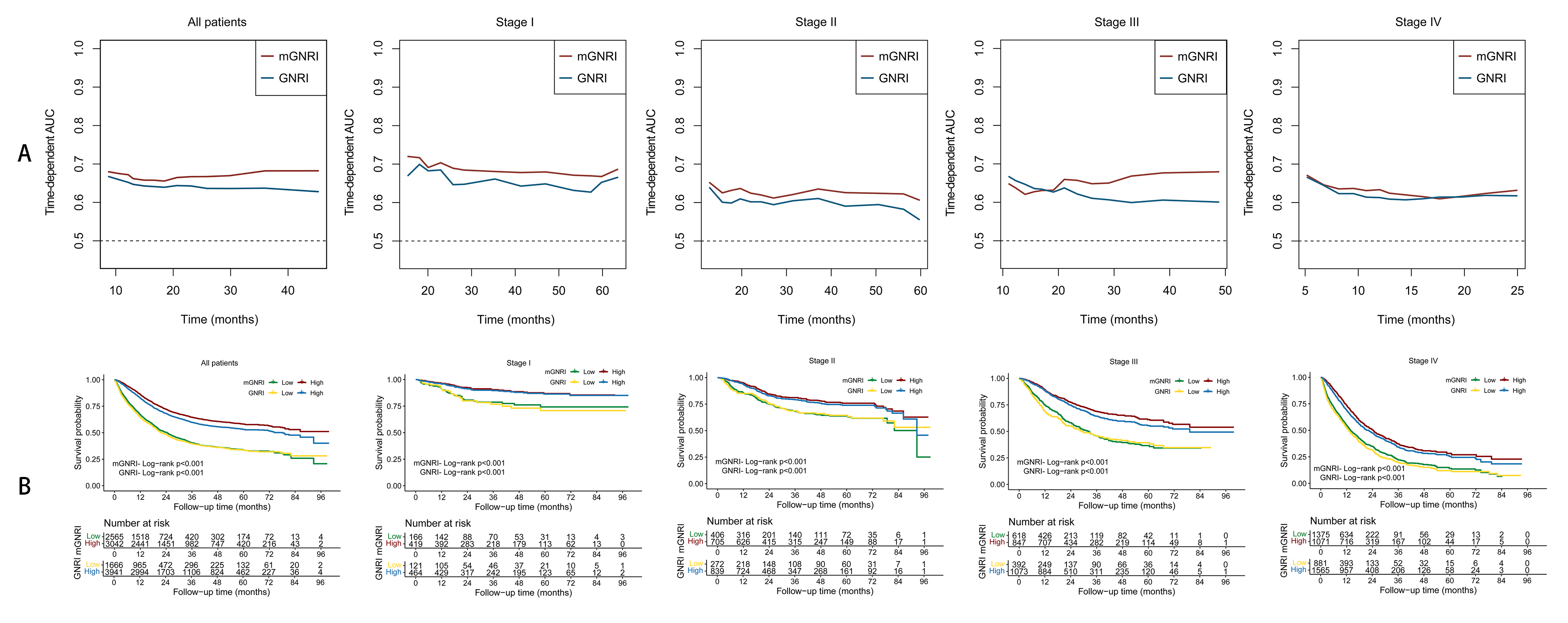

Supplement: Supplementary Figure S3 — Comparison the effectiveness of mGNRI and GNRI in predicting the prognosis of cancer patients. (A) The AUC of mGNRI and GNRI. (B) The survival curve of mGNRI and GNRI. [file Image_3.TIF]

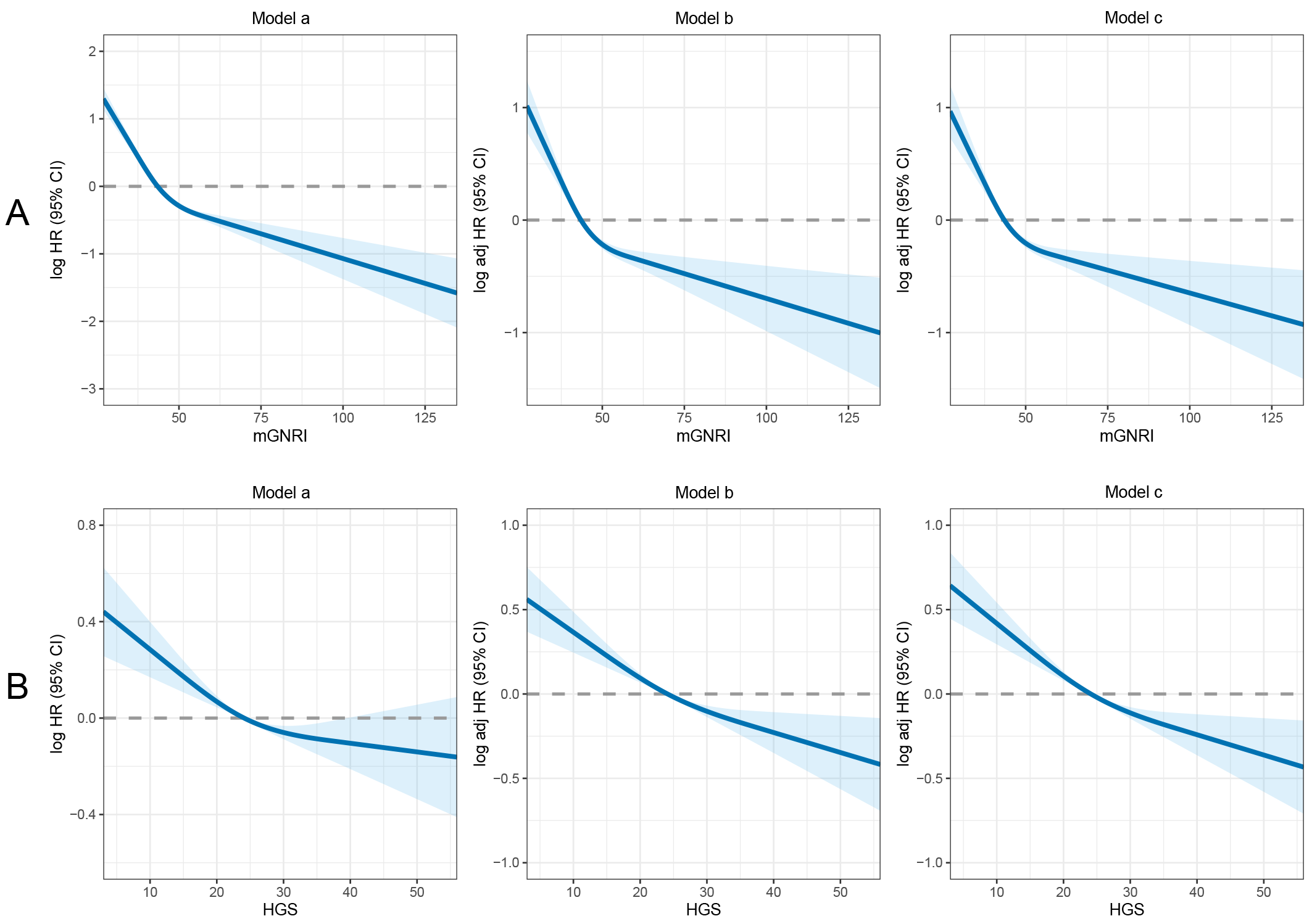

Supplement: Supplementary Figure S4 — The association between mGNRI and HGS and all-cause mortality in patients with cancer. (A), Mgnri; (B), HGS. Model a: No adjusted. Model b: Adjusted for age, sex, BMI, TNM stage. Model c: Adjusted for age, sex, BMI, TNM stage, tumor type, surgery, radiotherapy, chemotherapy, hypertension, diabetes, smoking, drinking, family history. [file Image_4.TIF]

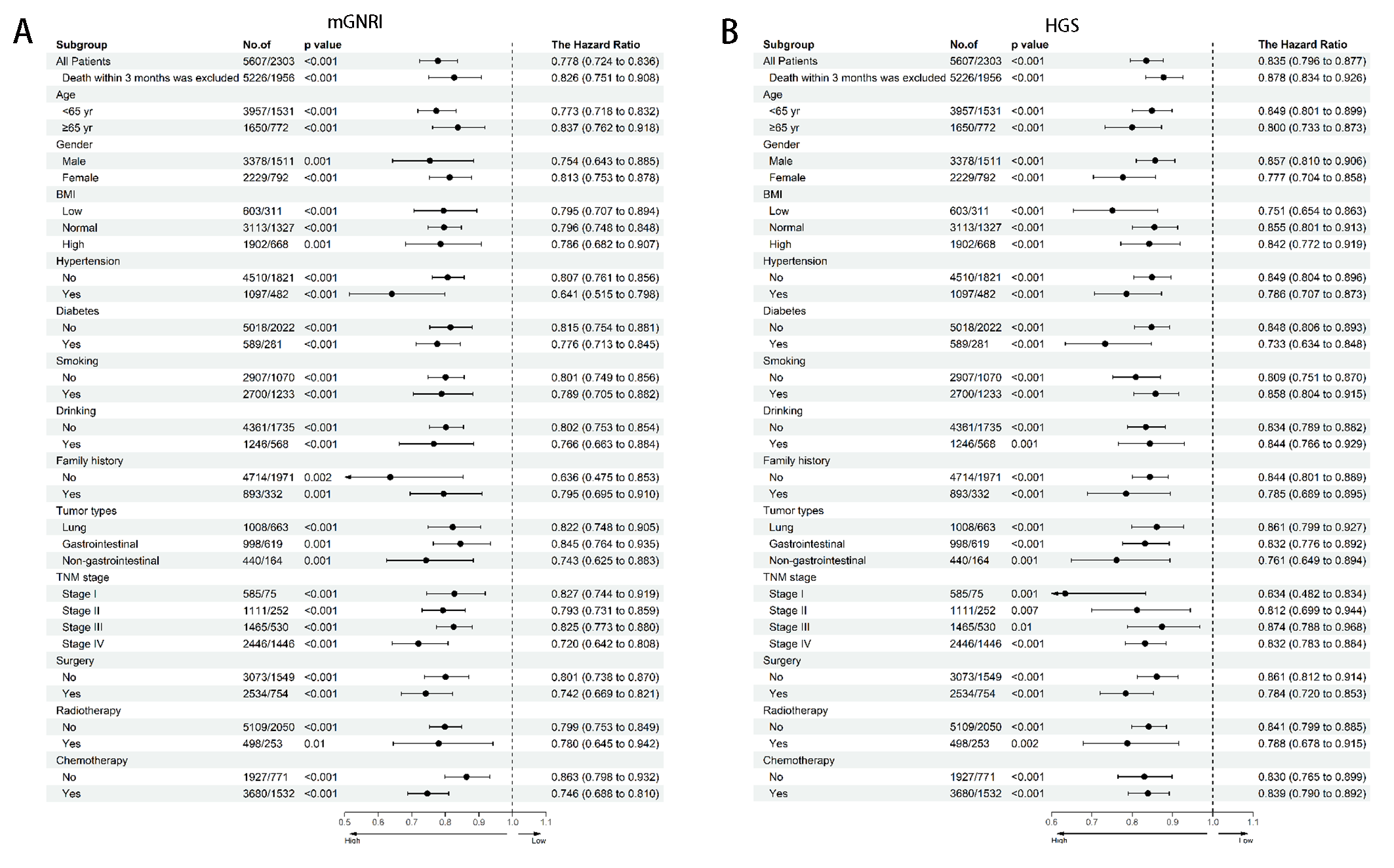

Supplement: Supplementary Figure S5 — The association between mGNRI and HGS and hazard risk of overall survival in various subgroups. (The model adjusted for age, sex, BMI, TNM stage, tumor type, surgery, radiotherapy, chemotherapy, hypertension, diabetes, smoking, drinking, family history). (A), mGNRI; (B), HGS. [file Image_5.TIF]

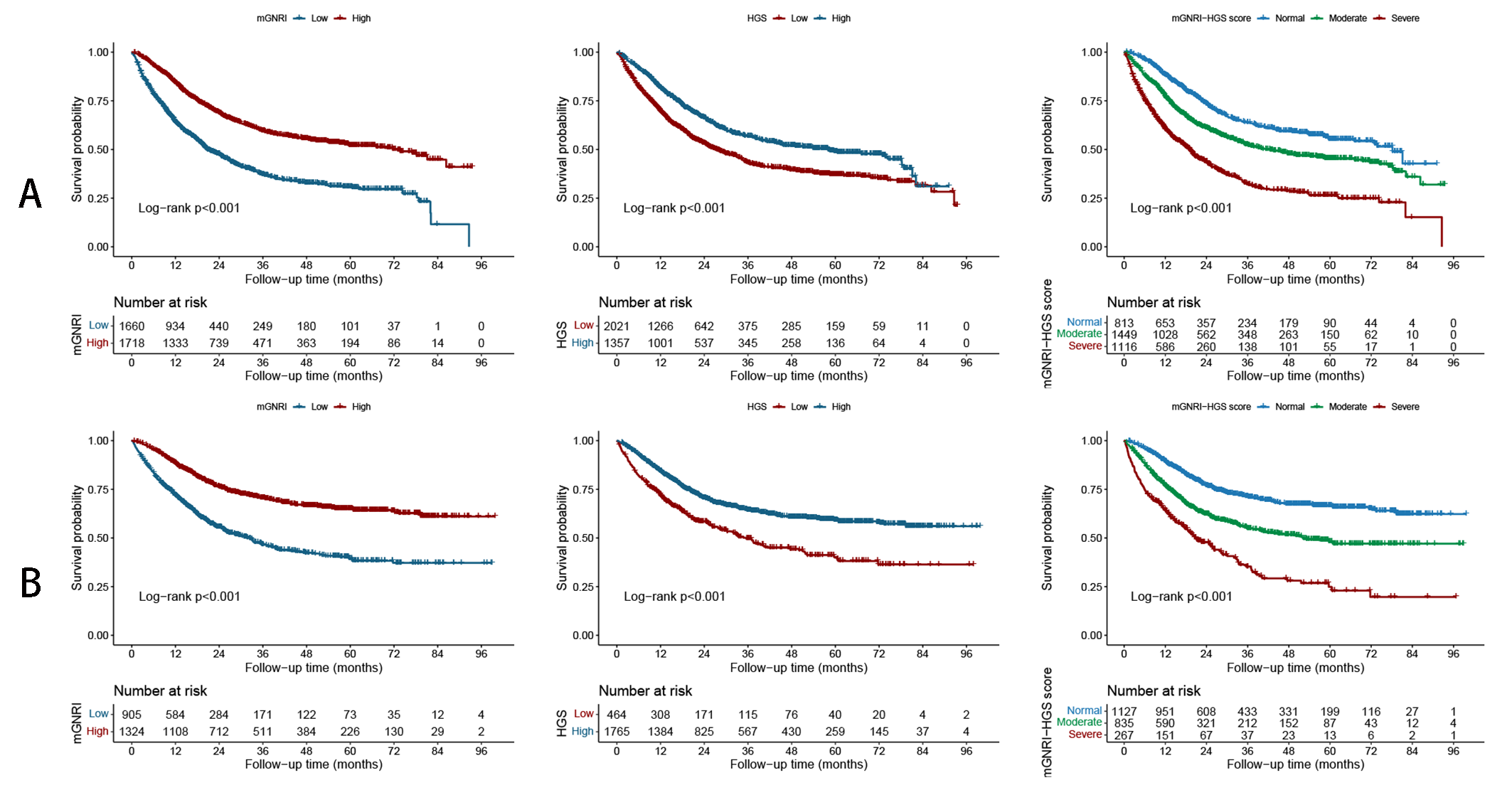

Supplement: Supplementary Figure S6 — Stratified survival analysis of mGNRI, HGS, and mGNRI-HGS score based on sex. (A), male; (B), female. [file Image_6.TIF]

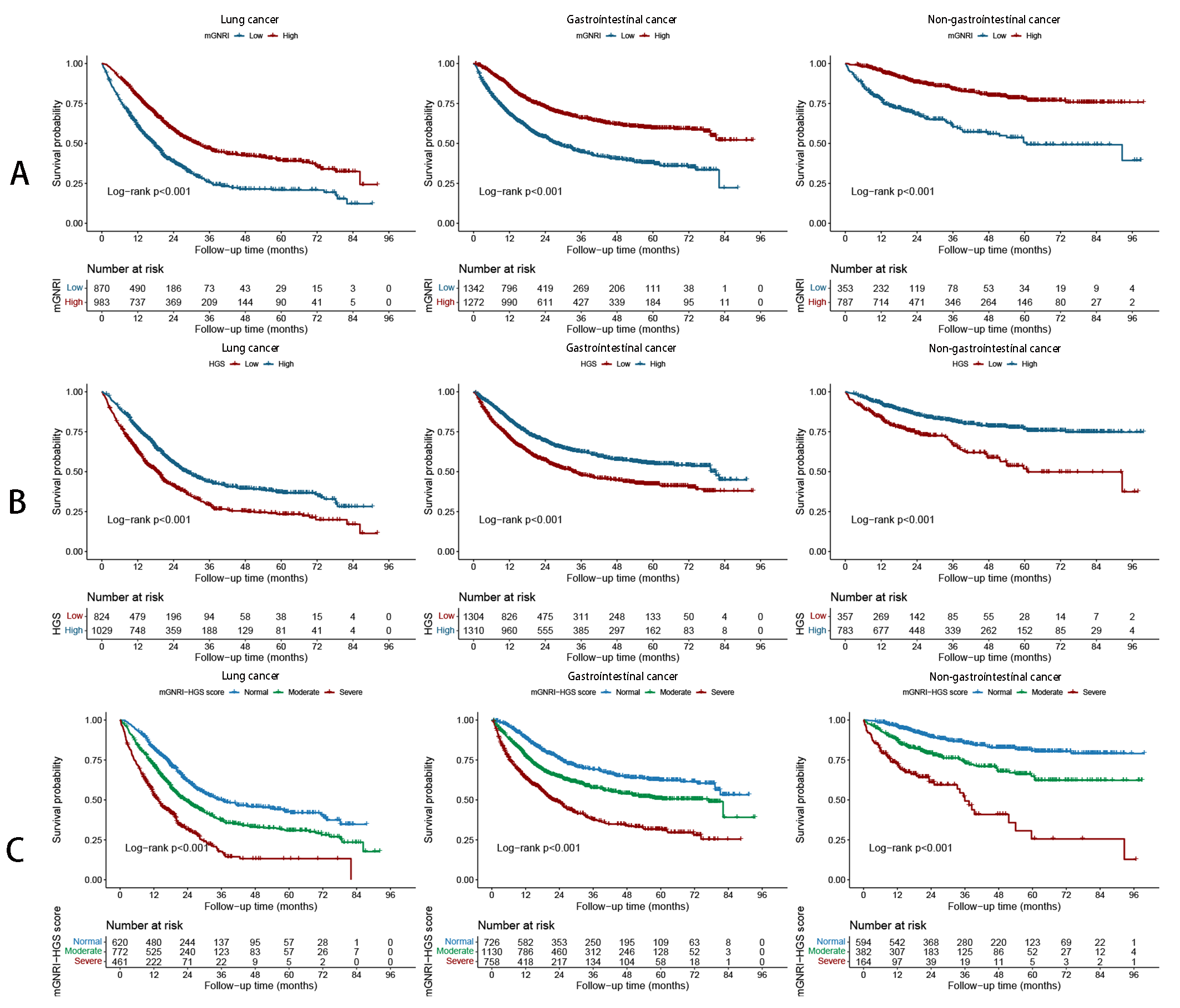

Supplement: Supplementary Figure S7 — Stratified survival analysis of mGNRI, HGS, and mGNRI-HGS score based on tumor types. (A), mGNRI; (B), HGS; (C), mGNRI-HGS score. [file Image_7.TIF]

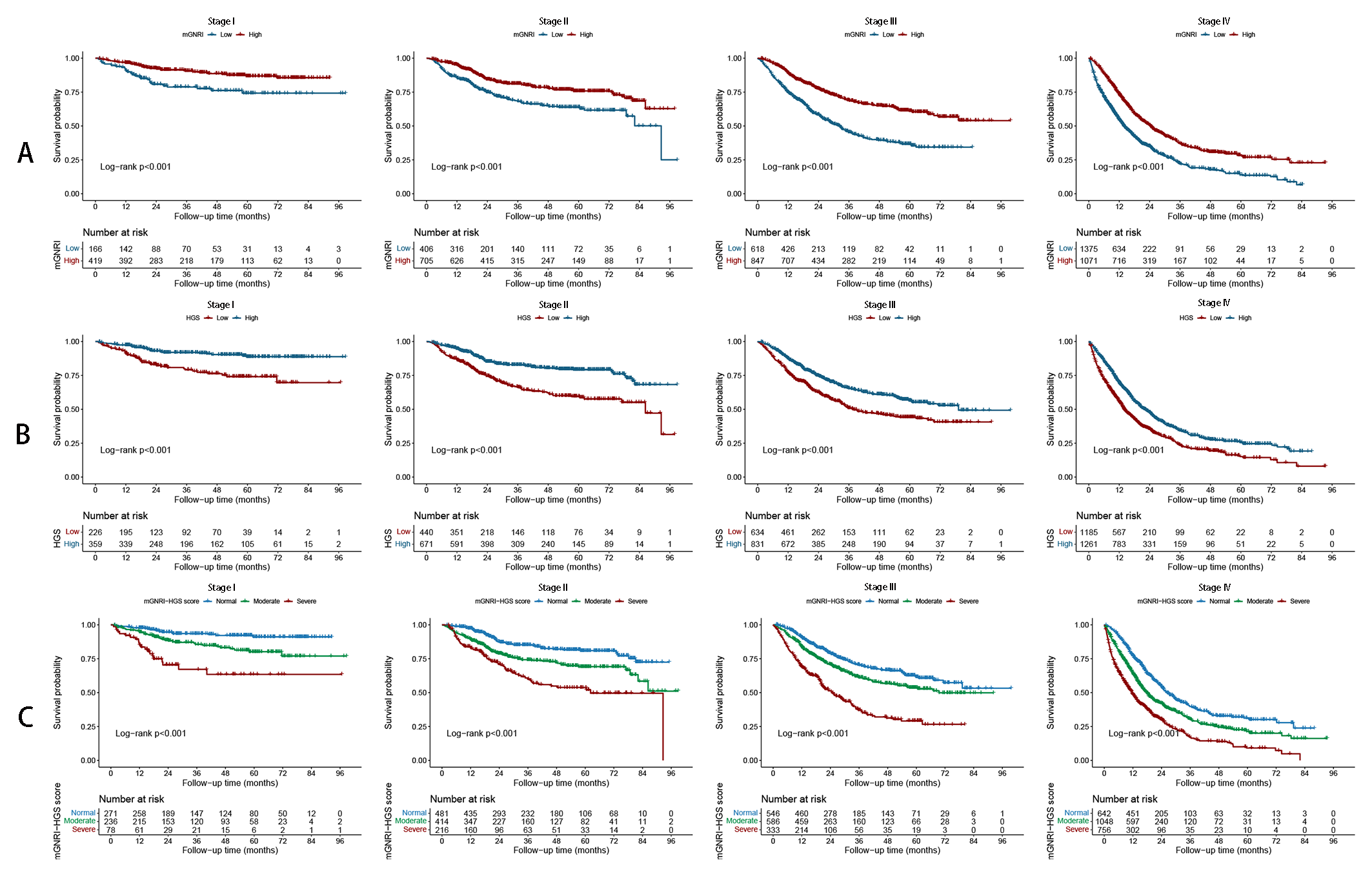

Supplement: Supplementary Figure S8 — Stratified survival analysis of mGNRI, HGS, and mGNRI-HGS score based on pathological stages. (A), mGNRI; (B), HGS; (C), mGNRI-HGS score. [file Image_8.TIF]
