# Supplementary material for: Association of Modified Geriatric Nutrition Risk Index and Handgrip Strength With Survival in Cancer: A Multi-Centre Cohort Study
Source: Front Nutr. 2022 Apr 1;9:850138. doi: 10.3389/fnut.2022.850138 (PMC9012584; doi:10.3389/fnut.2022.850138)
Supplement: Supplementary Table S1 — Association between the mGNRI, HGS and clinical characteristics. [file Table_1.DOCX]

**Table S1.** Association between the mGNRI, HGS and clinical characteristics.

| Characteristic | Overall  n=5607 | mGNRI | | | HGS | | |
| --- | --- | --- | --- | --- | --- | --- | --- |
|  |  | Low n=2565 | High n=3042 | p value | Low n=2485 | High n=3122 | p value |
| Sex, male, n (%) | 3378 (60.2) | 1660 (64.7) | 1718 (56.5) | <0.001 | 2021 (81.3) | 1357 (43.5) | <0.001 |
| Age, years, mean (SD) | 59.36 (11.25) | 60.00 (11.49) | 58.82 (11.02) | <0.001 | 63.11 (10.80) | 56.38 (10.69) | <0.001 |
| BMI, kg/m2, mean (SD) | 22.57 (4.54) | 20.76 (4.16) | 23.73 (3.68) | <0.001 | 21.87 (4.49) | 23.16 (4.54) | <0.001 |
| Hypertension, yes, n (%) | 1097 (19.6) | 489 (19.1) | 608 (20.0) | 0.404 | 528 (21.2) | 569 (18.2) | 0.005 |
| Diabetes, yes, n (%) | 589 (10.5) | 272 (10.6) | 317 (10.4) | 0.858 | 297 (12.0) | 292 ( 9.4) | 0.002 |
| Smoking yes, n (%) | 2700 (48.2) | 1383 (53.9) | 1317 (43.3) | <0.001 | 1518 (61.1) | 1182 (37.9) | <0.001 |
| Drinking, yes, n (%) | 1246 (22.2) | 635 (24.8) | 611 (20.1) | <0.001 | 696 (28.0) | 550 (17.6) | <0.001 |
| Family history, yes, n (%) | 893 (15.9) | 370 (14.4) | 523 (17.2) | 0.005 | 351 (14.1) | 542 (17.4) | 0.001 |
| Lung cancer, yes, n (%) | 1853 (33.0) | 870 (33.9) | 983 (32.3) | 0.214 | 824 (33.2) | 1029 (33.0) | 0.897 |
| Gastric cancer, yes, n (%) | 833 (14.9) | 467 (18.2) | 366 (12.0) | <0.001 | 435 (17.5) | 398 (12.7) | <0.001 |
| Esophagus cancer, yes, n (%) | 300 ( 5.4) | 163 (6.4) | 137 (4.5) | 0.003 | 194 ( 7.8) | 106 ( 3.4) | <0.001 |
| Hepatic-biliary cancer, yes, n (%) | 242 ( 4.3) | 113 (4.4) | 129 (4.2) | 0.813 | 122 ( 4.9) | 120 ( 3.8) | 0.059 |
| Pancreatic cancer, yes, n (%) | 134 ( 2.4) | 92 (3.6) | 42 (1.4) | <0.001 | 71 ( 2.9) | 63 ( 2.0) | 0.05 |
| Colorectal cancer, yes, n (%) | 1105 (19.7) | 507 (19.8) | 598 (19.7) | 0.946 | 482 (19.4) | 623 (20.0) | 0.625 |
| Gynecological cancer, yes, n (%) | 211 ( 3.8) | 96 (3.7) | 115 (3.8) | 0.997 | 44 ( 1.8) | 167 ( 5.3) | <0.001 |
| Urologic cancer, yes, n (%) | 197 ( 3.5) | 62 (2.4) | 135 (4.4) | <0.001 | 129 ( 5.2) | 68 ( 2.2) | <0.001 |
| Nasopharynx cancer, yes, n (%) | 119 ( 2.1) | 29 (1.1) | 90 (3.0) | <0.001 | 46 ( 1.9) | 73 ( 2.3) | 0.244 |
| Breast cancer, yes, n (%) | 486 ( 8.7) | 82 (3.2) | 404 (13.3) | <0.001 | 71 ( 2.9) | 415 (13.3) | <0.001 |
| Other cancer, yes, n (%) | 127 ( 2.3) | 84 (3.3) | 43 (1.4) | <0.001 | 67 ( 2.7) | 60 ( 1.9) | 0.065 |
| TNM stage, n (%) |  |  |  | <0.001 |  |  | <0.001 |
| Stage I | 585 (10.4) | 166 (6.5) | 419 (13.8) |  | 226 ( 9.1) | 359 (11.5) |  |
| Stage II | 1111 (19.8) | 406 (15.8) | 705 (23.2) |  | 440 (17.7) | 671 (21.5) |  |
| Stage III | 1465 (26.1) | 618 (24.1) | 847 (27.8) |  | 634 (25.5) | 831 (26.6) |  |
| Stage IV | 2446 (43.6) | 1375 (53.6) | 1071 (35.2) |  | 1185 (47.7) | 1261 (40.4) |  |
| WBC (median (IQR)) | 6.06 (3.07) | 6.82 (3.91) | 5.63 (2.52) | <0.001 | 6.31 (3.29) | 5.90 (2.91) | <0.001 |
| Neutrophil (mean (SD)) | 3.77 (2.78) | 4.59 (3.65) | 3.35 (2.06) | <0.001 | 4.09 (3.06) | 3.55 (2.58) | <0.001 |
| Lymphocyte (mean (SD)) | 1.47 (0.82) | 1.39 (0.86) | 1.53 (0.80) | <0.001 | 1.40 (0.81) | 1.52 (0.83) | <0.001 |
| PLT (median (IQR)) | 220.00 (111.00) | 239.00 (125.00) | 208.00 (95.00) | <0.001 | 218.00 (117.00) | 222.50 (104) | 0.013 |
| RBC (median (IQR)) | 4.27 (0.84) | 4.12 (0.89) | 4.38 (0.76) | <0.001 | 4.21 (0.91) | 4.31 (0.77) | <0.001 |
| Hb (median (IQR)) | 127.00 (27.00) | 120.00 (30.00) | 131.00 (24.00) | <0.001 | 125.00 (29.00) | 128.00 (25.00) | <0.001 |
| Albumin (mean (SD)) | 39.20 (6.8) | 36.60 (6.9) | 40.70 (5.5) | <0.001 | 37.90 (7.10) | 40.00 (6.00) | <0.001 |
| CRP (median (IQR)) | 4.08 (15.91) | 21.50 (43.53) | 3.00 (2.6) | <0.001 | 6.09 (25.98) | 3.28 (9.68) | <0.001 |
| KPS (median (IQR)) | 90.00 (10.00) | 90.00 (10.00) | 70.00 (10.00) | <0.001 | 90.00 (10.00) | 70.00 (10.00) | <0.001 |
| PG.SGA (median (IQR)) | 5.00 (6.00) | 7.00 (7.00) | 3.00 (4.00) | <0.001 | 6.00 (7.00) | 4.00 (5.00) | <0.001 |
| Cachexia, yes (%) | 1784 (31.8) | 1149 (44.8) | 635 (20.9) | <0.001 | 962 (38.7) | 822 (26.3) | <0.001 |
| Status (%) | 2303 (41.1) | 1328 (51.8) | 975 (32.1) | <0.001 | 1217 (49.0) | 1086 (34.8) | <0.001 |
| LOS (median (IQR)) | 10.00 (9.00) | 11.00 (9.00) | 10.00 (9.00) | <0.001 | 11.00 (10.00) | 10.00 (10.00) | <0.001 |
