# Supplementary material for: Association of Modified Geriatric Nutrition Risk Index and Handgrip Strength With Survival in Cancer: A Multi-Centre Cohort Study
Source: Front Nutr. 2022 Apr 1;9:850138. doi: 10.3389/fnut.2022.850138 (PMC9012584; doi:10.3389/fnut.2022.850138)
Supplement: Supplementary Table S2 — Cox regression analysis of characteristics associated with overall survival. [file Table_2.DOCX]

**Table S2.** Cox regression analysis of characteristics associated with overall survival.

| Characteristics | Univariate analysis | | Multivariate analysis | |
| --- | --- | --- | --- | --- |
|  | HR, 95% CI | p value | HR, 95% CI | p value |
| Sex, male | 0.684 (0.628-0.746) | <0.001 | 0.728 (0.668 - 0.794) | <0.001 |
| Age | 1.016 (1.013-1.02) | <0.001 | 1.004 (1 - 1.008) | 0.026 |
| BMI | 0.936 (0.924-0.947) | <0.001 | 0.992 (0.98 - 1.004) | 0.172 |
| Diabetes, yes | 1.271 (1.122-1.44) | <0.001 | 1.192 (1.052 - 1.352) | 0.006 |
| Hypertension, yes | 1.142 (1.033-1.263) | 0.009 | 1.041 (0.942 - 1.152) | 0.431 |
| Family history, yes | 0.836 (0.744-0.939) | 0.003 | 0.874 (0.778 - 0.982) | 0.023 |
| Smoking yes | 1.409 (1.298-1.53) | <0.001 | 1.131 (1.042 - 1.228) | 0.003 |
| Drinking, yes | 1.194 (1.086-1.312) | <0.001 | 0.993 (0.903 - 1.092) | 0.883 |
| TNM stage |  |  |  |  |
| Stage I | ref |  |  |  |
| Stage II | 1.93 (1.492-2.498) | <0.001 | 1.661 (1.455 - 1.896) | <0.001 |
| Stage III | 3.662 (2.874-4.665) | <0.001 | 2.746 (2.49 - 3.028) | <0.001 |
| Stage IV | 8.89 (7.039-11.226) | <0.001 | 5.575 (5.113 - 6.078) | <0.001 |
| Surgery, yes | 0.469 (0.43-0.512) | <0.001 | 0.603 (0.553 - 0.659) | <0.001 |
| Chemotherapy, yes | 1.114 (1.022-1.215) | 0.015 | 1.215 (1.114 - 1.325) | <0.001 |
| Radiotherapy, yes | 1.354 (1.189-1.543) | <0.001 | 0.959 (0.841 - 1.093) | 0.529 |
| Hb | 0.992 (0.99-0.994) | <0.001 | 0.996 (0.995 - 0.998) | <0.001 |
| WBC | 1.001 (1-1.002) | 0.006 | 1.001 (1 - 1.002) | 0.169 |
| Neutrophil | 1.004 (1.002-1.006) | <0.001 | 1.001 (0.998 - 1.004) | 0.599 |
| Lymphocyte | 0.998 (0.988-1.009) | 0.773 |  |  |
| RBC | 1.001 (1-1.001) | 0.001 | 1.001 (1.001 - 1.001) | <0.001 |
| PLT | 1.002 (1.001-1.002) | <0.001 | 1.001 (1 - 1.001) | 0.005 |
| Albumin | 0.953 (0.947-0.96) | <0.001 | 0.994 (0.988 - 1) | 0.063 |
| HGS | 0.988 (0.983-0.992) | <0.001 | 0.993 (0.989 - 0.997) | 0.001 |
| mGNRI | 0.962 (0.957-0.967) | <0.001 | 0.986 (0.981 - 0.99) | <0.001 |
| KPS | 0.975 (0.973-0.978) | <0.001 | 0.987 (0.985 - 0.99) | <0.001 |
| PGSGA | 1.088 (1.079-1.096) | <0.001 | 1.032 (1.024 - 1.04) | <0.001 |
