# Supplementary material for: Association of Modified Geriatric Nutrition Risk Index and Handgrip Strength With Survival in Cancer: A Multi-Centre Cohort Study
Source: Front Nutr. 2022 Apr 1;9:850138. doi: 10.3389/fnut.2022.850138 (PMC9012584; doi:10.3389/fnut.2022.850138)
Supplement: Supplementary Table S3 — Trend test of the relationship between mGNRI and HGS and survival. [file Table_3.DOCX]

**Table S3.** Trend test of the relationship between mGNRI and HGS and survival.

| mGNRI | Model a | p value | Model b | p value | Model b | p value |
| --- | --- | --- | --- | --- | --- | --- |
| Continuous (per SD) | 0.670 (0.630,0.713) | <0.001 | 0.783 (0.739,0.829) | <0.001 | 0.797 (0.753,0.844) | <0.001 |
| Cutoff value |  | <0.001 |  | <0.001 |  | <0.001 |
| C1 | ref |  | ref |  | ref |  |
| C2 | 0.455 (0.419,0.494) |  | 0.592 (0.541,0.648) |  | 0.623 (0.569,0.683) |  |
| Quartiles |  |  |  |  |  |  |
| Q1 | ref |  | ref |  | ref |  |
| Q2 | 0.793 (0.715,0.88) | <0.001 | 0.922 (0.811,1.048) | 0.216 | 0.934 (0.821,1.062) | 0.296 |
| Q3 | 0.482 (0.429,0.541) | <0.001 | 0.625 (0.541,0.721) | <0.001 | 0.668 (0.577,0.772) | <0.001 |
| Q4 | 0.348 (0.308,0.392) | <0.001 | 0.515 (0.448,0.591) | <0.001 | 0.545 (0.474,0.626) | <0.001 |
| p for trend |  | <0.001 |  | <0.001 |  | <0.001 |
| HGS | Model a | p value | Model b | p value | Model b | p value |
| Continuous (per SD) | 0.895 (0.86,0.931) | <0.001 | 0.85 (0.81,0.891) | <0.001 | 0.835 (0.796,0.877) | <0.001 |
| Cutoff value |  |  |  |  |  |  |
| C1 | ref |  | ref |  | ref |  |
| C2 | 0.583 (0.537,0.633) | <0.001 | 0.739 (0.674,0.812) | <0.001 | 0.719 (0.655,0.79) | <0.001 |
| p for trend |  | <0.001 |  | <0.001 |  | <0.001 |
| Quartiles |  |  |  |  |  |  |
| Q1 | ref |  | ref |  | ref |  |
| Q2 | 0.789 (0.704,0.884) | <0.001 | 0.790 (0.704,0.887) | <0.001 | 0.761 (0.678,0.855) | <0.001 |
| Q3 | 0.876 (0.783,0.979) | 0.020 | 0.754 (0.667,0.853) | <0.001 | 0.751 (0.663,0.85) | <0.001 |
| Q4 | 0.740 (0.659,0.830) | <0.001 | 0.618 (0.538,0.71) | <0.001 | 0.587 (0.511,0.675) | <0.001 |
| p for trend |  | <0.001 |  | 0.001 |  | <0.001 |

Notes:

Model a: No adjusted.

Model b: Adjusted for age, sex, BMI, TNM stage.

Model c: Adjusted for age, sex, BMI, TNM stage, tumor type, surgery, radiotherapy, chemotherapy, hypertension, diabetes, smoking, drinking, family history.
