# Supplementary material for: Association of Modified Geriatric Nutrition Risk Index and Handgrip Strength With Survival in Cancer: A Multi-Centre Cohort Study
Source: Front Nutr. 2022 Apr 1;9:850138. doi: 10.3389/fnut.2022.850138 (PMC9012584; doi:10.3389/fnut.2022.850138)
Supplement: Supplementary Table S4 — Logistic regression analysis of mGNRI and HGS associated with secondary outcome. [file Table_4.DOCX]

**Table S4.** Logistic regression analysis of mGNRI and HGS associated with secondary outcome.

| Factors | Model a | p value | Model b | p value | Model c | p value |
| --- | --- | --- | --- | --- | --- | --- |
| KPS | | | | | | |
| mGNRI | 0.945 (0.934,0.956) | <0.001 | 0.970 (0.959,0.981) | <0.001 | 0.971 (0.960,0.981) | <0.001 |
| HGS | 0.930 (0.921,0.939) | <0.001 | 0.925 (0.914,0.936) | <0.001 | 0.927 (0.916,0.938) | <0.001 |
| PGSGA | | | | | | |
| mGNRI | 0.967 (0.962,0.972) | <0.001 | 0.978 (0.973,0.983) | <0.001 | 0.978 (0.973,0.983) | <0.001 |
| HGS | 0.969 (0.963,0.974) | <0.001 | 0.965 (0.958,0.972) | <0.001 | 0.965 (0.957,0.972) | <0.001 |
| Cachexia | | | | | | |
| mGNRI | 0.962 (0.956,0.969) | <0.001 | 0.986 (0.981,0.992) | <0.001 | 0.986 (0.981,0.992) | <0.001 |
| HGS | 0.973 (0.967,0.979) | <0.001 | 0.977 (0.969,0.985) | <0.001 | 0.977 (0.969,0.985) | <0.001 |
| Admission 90 days outcome | | | | | | |
| mGNRI | 0.903 (0.886,0.920) | <0.001 | 0.942 (0.921,0.963) | <0.001 | 0.945 (0.924,0.966) | <0.001 |
| HGS | 0.954 (0.943,0.966) | <0.001 | 0.944 (0.930,0.958) | <0.001 | 0.947 (0.933,0.961) | <0.001 |

Notes:

Model a: No adjusted.

Model b: Adjusted for age, sex, BMI, TNM stage.

Model c: Adjusted for age, sex, BMI, TNM stage, tumor type, surgery, radiotherapy, chemotherapy, hypertension, diabetes, smoking, drinking, family history.
