# Supplementary material for: Association of Modified Geriatric Nutrition Risk Index and Handgrip Strength With Survival in Cancer: A Multi-Centre Cohort Study
Source: Front Nutr. 2022 Apr 1;9:850138. doi: 10.3389/fnut.2022.850138 (PMC9012584; doi:10.3389/fnut.2022.850138)
Supplement: Supplementary Table S5 — Association between the mGNRI-HGS score and clinical characteristics. [file Table_5.DOCX]

**Table S5.** Association between the mGNRI-HGS score and clinical characteristics.

| Characteristic | mGNRI-HGS score | | | p value |
| --- | --- | --- | --- | --- |
|  | Normal n=1940 | Moderate n=2284 | Severe n=1383 |  |
| Sex, male, n (%) | 813 (41.9) | 1449 (63.4) | 1116 (80.7) | <0.001 |
| Age, years, mean (SD) | 56.44 (10.53) | 59.52 (11.31) | 63.19 (10.95) | <0.001 |
| BMI, kg/m2, mean (SD) | 24.02 (3.85) | 22.43 (4.43) | 20.45 (4.03) | <0.001 |
| Hypertension, yes, n (%) | 358 (18.5) | 461 (20.2) | 278 (20.1) | 0.312 |
| Diabetes, yes, n (%) | 177 ( 9.1) | 255 (11.2) | 157 (11.4) | 0.049 |
| Smoking yes, n (%) | 677 (34.9) | 1145 (50.1) | 878 (63.5) | <0.001 |
| Drinking, yes, n (%) | 319 (16.4) | 523 (22.9) | 404 (29.2) | <0.001 |
| Family history, yes, n (%) | 366 (18.9) | 333 (14.6) | 194 (14.0) | <0.001 |
| Lung cancer, yes, n (%) | 620 (32.0) | 772 (33.8) | 461 (33.3) | 0.433 |
| Gastric cancer, yes, n (%) | 186 ( 9.6) | 392 (17.2) | 255 (18.4) | <0.001 |
| Esophagus cancer, yes, n (%) | 61 ( 3.1) | 121 ( 5.3) | 118 ( 8.5) | <0.001 |
| Hepatic-biliary cancer, yes, n (%) | 72 ( 3.7) | 105 ( 4.6) | 65 ( 4.7) | 0.266 |
| Pancreatic cancer, yes, n (%) | 24 ( 1.2) | 57 ( 2.5) | 53 ( 3.8) | <0.001 |
| Colorectal cancer, yes, n (%) | 383 (19.7) | 455 (19.9) | 267 (19.3) | 0.901 |
| Gynecological cancer, yes, n (%) | 99 ( 5.1) | 84 ( 3.7) | 28 ( 2.0) | <0.001 |
| Urologic cancer, yes, n (%) | 54 ( 2.8) | 95 ( 4.2) | 48 ( 3.5) | 0.053 |
| Nasopharynx cancer, yes, n (%) | 59 ( 3.0) | 45 ( 2.0) | 15 ( 1.1) | <0.001 |
| Breast cancer, yes, n (%) | 354 (18.2) | 111 ( 4.9) | 21 ( 1.5) | <0.001 |
| Other cancer, yes, n (%) | 28 ( 1.4) | 47 ( 2.1) | 52 ( 3.8) | <0.001 |
| TNM stage, n (%) |  |  |  |  |
| Stage I | 271 (14.0) | 236 (10.3) | 78 ( 5.6) | <0.001 |
| Stage II | 481 (24.8) | 414 (18.1) | 216 (15.6) |  |
| Stage III | 546 (28.1) | 586 (25.7) | 333 (24.1) |  |
| Stage IV | 642 (33.1) | 1048 (45.9) | 756 (54.7) |  |
| WBC (median (IQR)) | 5.59 (2.52) | 6.12 (3.01) | 7.06 (4.1) | <0.001 |
| Neutrophil (mean (SD)) | 3.30 (2.03) | 3.76 (2.78) | 4.78 (3.76) | <0.001 |
| Lymphocyte (mean (SD)) | 1.57 (0.79) | 1.46 (0.83) | 1.32 (0.84) | <0.001 |
| PLT (median (IQR)) | 212.00 (94.00) | 220.00 (111.00) | 234.00 (130.00) | <0.001 |
| RBC (median (IQR)) | 4.38 (0.74) | 4.28 (0.86) | 4.05 (0.90) | <0.001 |
| Hb (median (IQR)) | 131.00 (23.00) | 127.00 (28.00) | 119.00 (30.50) | <0.001 |
| Albumin (mean (SD)) | 41.00 (5.2) | 39.20 (6.52) | 35.80 (7.00) | <0.001 |
| CRP (median (IQR)) | 3.02 (2.53) | 4.89 (16.8) | 24.50 (46.85) | <0.001 |
| KPS (median (IQR)) | 90.00 (10.00) | 90.00 (10.00) | 80.00 (10.00) | <0.001 |
| PG.SGA (median (IQR)) | 3.00 (5.00) | 5.00 (6.00) | 8.00 (7.00) | <0.001 |
| Cachexia, yes (%) | 350 (18.0) | 757 (33.1) | 677 (49.0) | <0.001 |
| Status (%) | 553 (28.5) | 955 (41.8) | 795 (57.5) | <0.001 |
| LOS (median (IQR)) | 10.00 (9.00) | 10.00 (10.00) | 12.00 (9.00) | <0.001 |
