# Supplementary material for: Association of Modified Geriatric Nutrition Risk Index and Handgrip Strength With Survival in Cancer: A Multi-Centre Cohort Study
Source: Front Nutr. 2022 Apr 1;9:850138. doi: 10.3389/fnut.2022.850138 (PMC9012584; doi:10.3389/fnut.2022.850138)
Supplement: Supplementary Table S6 — Comparative analysis of the discrimination of mGNRI, HGS, mGNRI-HGS score for all-cause mortality in patients with cancer. [file Table_6.DOCX]

**Table S6.** Comparative analysis of the discrimination of mGNRI, HGS, mGNRI-HGS score for all-cause mortality in patients with cancer.

| Discrimination Ability | C-statistic | | cNRI | | IDI | |
| --- | --- | --- | --- | --- | --- | --- |
|  | Difference | p value | Difference | p value | Difference | p value |
| mGNRI-HGS score | Ref |  | Ref |  | Ref |  |
| mGNRI | -0.021(-0.028,-0.013) | <0.001 | -0.179(-0.222, -0.134) | <0.001 | -0.016(-0.028, -0.003) | 0.020 |
| HGS | -0.054(-0.062, -0.048) | <0.001 | -0.259(-0.302, 0.217) | <0.001 | -0.053(-0.065, -0.041) | <0.001 |
| Model performance after the addition of other indexes to the TNM stage for predicting all-cause mortality | | | | | | |
| Model | C-statistic | P value | cNRI | p value | IDI | p value |
| TNM stage | 0.679(0.669,0.689) | <0.001 | Ref |  | Ref |  |
| TNM stage + mGNRI-HGS score | 0.719(0.708,0.729) | <0.001 | 0.227(0.010,0.271) | 0.030 | 0.035(0.016,0.036) | <0.001 |
| TNM stage + mGNRI | 0.707(0.697,0.717) | <0.001 | 0.259(0.215,0.300) | <0.001 | 0.025(0.016,0.036) | <0.001 |
| TNM stage + HGS | 0.701(0.691,0.716) | <0.001 | 0.179(0.135,0.228) | <0.001 | 0.015(0.008,0.023) | <0.001 |

Table note: cNRI, continuous net reclassification improvement; IDI, integrated discrimination improvement.
